# ADFAC: Automatic detection of facial articulatory features

**DOI:** 10.1016/j.mex.2020.101006

**Published:** 2020-07-22

**Authors:** Saurabh Garg, Ghassan Hamarneh, Allard Jongman, Joan A. Sereno, Yue Wang

**Affiliations:** a Language and Brain Lab, Department of Linguistics, Simon Fraser University, Canada; b Medical Image Analysis Lab, Simon Fraser University, Canada; c KU Phonetics and Psycholinguistics Lab, Department of Linguistics, University of Kansas, United States

**Keywords:** Visual cues, Facial movements, Features, Discriminative analysis, Computer vision, Image processing, Machine learning

## Abstract

Using computer-vision and image processing techniques, we aim to identify specific visual cues as induced by facial movements made during monosyllabic speech production. The method is named ADFAC: Automatic Detection of Facial Articulatory Cues. Four facial points of interest were detected automatically to represent head, eyebrow and lip movements: nose tip (proxy for head movement), medial point of left eyebrow, and midpoints of the upper and lower lips. The detected points were then automatically tracked in the subsequent video frames. Critical features such as the distance, velocity, and acceleration describing local facial movements with respect to the resting face of each speaker were extracted from the positional profiles of each tracked point. In this work, a variant of random forest is proposed to determine which facial features are significant in classifying speech sound categories. The method takes in both video and audio as input and extracts features from any video with a plain or simple background. The method is implemented in MATLAB and scripts are made available on GitHub for easy access.

• Using innovative computer-vision and image processing techniques to automatically detect and track keypoints on the face during speech production in videos, thus allowing more natural articulation than previous sensor-based approaches.
• Measuring multi-dimensional and dynamic facial movements by extracting time-related, distance-related and kinematics-related features in speech production.
• Adopting the novel random forest classification approach to determine and rank the significance of facial features toward accurate speech sound categorization.

## Graphical abstract

**Figure:**
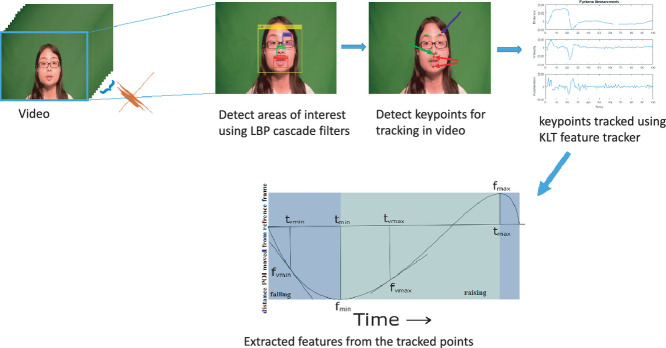

Specifications Table

**Table:** 

Subject Area	Computer Science
More specific subject area	Linguistics /Phonetics/ Speech Science
Method name	ADFAC
Name and reference of original method	Garg, S.; Hamarneh, G.; Jongman, A.; Sereno, J.A.; Wang, Y. Computer-vision analysis reveals facial movements made during Mandarin tone production align with pitch trajectories. Speech Communication. 2019, 113, 47–62.
Resource availability	https://github.com/srbhgarg/avc.git

## Introduction

### Background

Different research articles have reported different methods to acquire and analyze visual speech articulatory movement data. In one commonly used method (e.g., [6]), human annotators watch a video recording of a speaker and then annotate the facial speech articulatory movements. This method has several limitations that make them unsuitable for replication in a different lab. For example, the movements observed by the human annotators are limited by their attention field, and thus are judged in a subjective manner. Moreover, the observed movements are only limited to the number of occurrences of a particular event (e.g., number of times head moved up and down), whereas quantitative measurements such as the intensity, magnitude, and temporal information of the motion are not possible.

Sensor-based approaches are considered more quantifiable and precise in measuring and tracking the movements than human annotators. A variety of sensor-based methods are reported in the literature such as the use of electromagnetic (electromagnetic articulography, EMA) and optical (OPTOTRAK) motion tracking systems. For example, the study of tone-vowel co-production by Shaw et al. [17] used EMA. This method involves putting sensor coils on the various parts of a speaker's face and mouth, including lips, tongue, and jaw. An induction current is induced in the sensor coils by external coils placed around the head. The amount of current induced can be used to estimate the position of the sensor and its movement over time, thus making it possible to measure the spatial variations of articulatory displacement and distance with millimeter precision. Additionally, a number of studies (e.g., [1,^2^,10]) used the OPTOTRAK (Northern Digital Inc.) system. This system involves putting infrared emitting sensors/markers on various locations of the head and face. For example, OPTOTRAK has been used to capture eyebrow and jaw movements for sentence focus, with measurements of the displacement and peak velocity of these movements [10]. Another similar sensor-based method using the motion capture system involves attaching retro-reflectors (Qualisys AB) to the speaker's face for recording, allowing analysis of lip, eyebrow, and head displacement magnitude and movement velocity [16]. These sensor-based systems have limitations of their own. The placement of actual physical sensors on the speaker's face or tongue may interfere with the natural facial movements and may cause discomfort over long durations. Furthermore, although sensor-based methods are more precise in capturing motion than annotation-based methods, only limited regions where the sensors are placed can be analyzed.

### The current method

Our proposed method (ADFAC) tackles the current limitations in articulatory measurements by making the measurements free from any sensor placement so that the articulations can occur in natural settings without any discomfort to the speakers. In this paper, recent advancements in computer vision and image processing techniques are leveraged to propose an automatic way to quantify various movements of keypoints on the face. Our method has the advantage that it does not involve any human annotators or placement of any sensors. Furthermore, with the proposed method, different feature types and dimensions such as distance-based, time-based and kinematics-based can be extracted from the articulatory movements recorded in the video. These features are useful in understanding speech articulation and perception.

ADFAC can be used in several under-explored research areas for understanding the role of visual cues in speech production. Firstly, it is unclear which specific visual cues reflect individual linguistic features. Since previous research employed a variety of data acquisition and analysis techniques and focused on different facial regions, there is a lack of consistency and comparability in the findings across studies.

The present method uses computer-vision and image-analysis techniques to systematically identify and examine the features extracted from motion captures of speakers’ productions of Mandarin tones in single words [8]. Mandarin tone is used as a test case in this study because the production of lexical tone, as a prosodic entity, has been claimed to involve additional visual cues (e.g., head and eyebrow movements) beyond mouth movement cues primarily associated with segmental speech production. Thus, our method captures a wide range of visual facial cues in speech production. The novelty of our core method lies in connecting all the building blocks (e.g. keypoint detection, tracking and feature extraction) together to create a functional pipeline. Further, the method is customized to provide more precise segmentation boundaries and to handle problematic cases by analyzing image frames using a holistic combination of heuristics.

In addition to tracking and feature extraction, we also proposed a novel analysis method based on random forest for classification task. Random forest has several advantages including that it can differentiate and quantify which features are important for a particular class, which has not been previously examined. In our analysis we used a variant of random forest that can also test the significance of each feature along with the feature importance.

## Method details

### Video analysis

The ADFAC was implemented in MATLAB. This fully automatic computer-vision based method is divided into the following steps: 1) Segmenting videos into separate tokens; 2) Detecting keypoints on the face such as the tip of the nose, cupid bow of the lips and the center point of the eyebrow; 3) Tracking keypoint over the duration of the utterance of a token; 4) Computing features from the tracked keypoints.

#### Segmenting speech tokens on video

In our collected data, multiple word tokens were recorded in a single video. So the first step is to segment each of the tokens into a separate video token file. This is done automatically with the help of the audio signal as suggested in Garg et al. [7]. Briefly, the method works by looking at the amplitude of the audio signal and wherever the magnitude goes over a certain threshold value (that was determined empirically and was set at 20% of maximum amplitude), the video signal is segmented. Anything lower than the determined threshold value is considered noise such as sounds of the keystrokes of the keyboard or cough and is subsequently removed from the video recordings, leaving behind the video recording of the token-word utterance. In order to accommodate segmentation-related errors, a fixed number of frames are added on either side of the segmented video. In our experiments, the video frame rate was 29.97 frames/s and the audio signal was sampled at 16 kHz. Additionally, a fixed buffer of 10 frames i.e. equivalent of 0.3 s of the video token is added. This step of video segmentation is optional if the data is already saved in separate word tokens instead of one continuous video.

#### Identifying regions-of-interest

Once the tokens are segmented, the video of a sample token is read frame by frame. On the first frame of the video, different regions of the face are identified that will be used to localize keypoints on the face. In the proposed method, focus is given specifically to three regions-of-interest (ROI); namely, the head, the eyebrows and the lips. These regions are sufficient in most articulatory studies but can be easily extended to other regions such as chin. To identify the regions, we used cascade filters as proposed by Lienhart et al. [11]. The output of a cascade filter is a bounding box around the ROI. Once a rough bounding box is obtained, part-specific detectors are then used to refine these localizations and to obtain keypoints from the bounding boxes. Next, we discuss these part-specific detectors in detail:

**Face**: In order to reduce the search space and make the detection of ROIs more reliable, the location of the face is detected first. The face detection is implemented using Local Binary Pattern (LBP) cascade filters [14]. In our recorded videos of human speakers, there is one face in each of the video frames. In case of multiple detections by the cascade filter, the bounding boxes are merged together to yield one box. The number of detections can be controlled by the merge-threshold parameter in MATLAB. In order to detect one bounding box, a predefined range of values for merge-threshold is used one by one until an output with just one bounding box is obtained. In the MATLAB implementation, the merge-threshold controls the number of bounding boxes where multiple detections around the area of interest are detected. As the name suggests, the co-located detections that meet the threshold value are merged to produce one bounding box around the target object. The larger the value of merge-threshold the more detections will be merged and fewer bounding boxes corresponding to the region of interest will be returned to the user. However, in real experiments, one fixed value does not always work so the best merge-threshold value is iteratively decided automatically. Merge-threshold is set to a low value and its value is incrementally increased until the function returns one bounding box corresponding to one face in the video. Once a face bounding box is obtained, all further searches are narrowed down within this box. Limiting the search space to the face region helped reduce a large number of false positive detections. In our experiments, merge threshold was varied from 1 to 150 with increments of 10.

**Head:** Since the nose lies at the center of the head and cannot move independently of the head, the movement of the nose is a good estimate of the movement of the head. Previous studies (e.g., [3,19]) have also used the nose as a marker for head movement. Further, the nose can be located by using a predefined LBP cascade filter in MATLAB. Again, the value of the merge-threshold is varied to get one bounding box. In order to track the movements of the head, one consistent point on the nose is needed that can be detected in different speaker videos. For this purpose, the nose tip is chosen as it lies on a well-defined edge. The lower edge of the nose is detected using an active contour model where initial contour is defined by the bounding box detected earlier and is subsequently refined iteratively. The smoothness of the contour is a parameter that can be controlled in MATLAB. In our experiments, an edge-based model with smoothness factor of 2 was used to evolve the contour at each iteration step. The contour was evolved up to a maximum of 500 iterations.

**Eyebrows:** Eyebrows are detected in two steps. In the first step, the eyes are located and in the second step the position of the eyebrows is found based on the information obtained from the first step. The eyes are again located using a set of LBP cascaded filters. MATLAB contains two different types of LBP filters: One that detects the location of the pair of eyes together and another set of filters that detect the left and right eye separately. Although we are interested in the eyes separately, our experiments show that detecting the eye pairs first is more robust with fewer false positives and immensely improves the detection of separate eyes. This is due to the fact that the individual eye bounding box should lie within or near the detected eye-pair bounding box. Again, merge-threshold is used to robustly detect the eye pairs bounding box. The merge threshold is varied from 1 to 150 and the increments are adaptively chosen based on the number of bounding boxes returned by the function. When we have a reliable eye-pair bounding box then left-eye specific filters are used to detect the left eye only. This left-eye specific filter returns multiple bounding boxes for the left eye which are then refined and selected based on the distance from the top left corner of the bounding box of the detected eye-pair to the left eye bounding box. The box with a minimum distance was selected. Once the bounding box around the eye is obtained, local information is used to refine the position of the eyebrows based on several different factors such as color and contour. Using the local information, the contour of the eyebrow (i.e. the superciliary ridge line) is extracted as described below.

1) Based on color: Since the eyebrows are darker in color, their pixel intensities are relatively smaller in value than the skin around it. This information is used to narrow down the location of the eyebrow. A patch of PxP pixels (*P* = 10% of the height of the detected eyebrow box) is compared to the adjacent patch below it. If the sum of pixels in a patch is larger than in the adjacent patch below it then that patch is considered a potential candidate for the eyebrow line. Similarly, every pixel inside the eye bounding box and adjacent area on top of the box is evaluated. Every pixel is then assigned a probability of it belonging to the eyebrow ridge line.
2) Based on edge: Since the skin is detected better in HSV space, the RGB image around the eye is first converted to HSV using rgb2hsv MATLAB function and then a Canny edge detector is used to determine the edges around the eyebrow. The detected edges in the top half of the box mark the area where the superciliary ridge line has the highest probability.
3) Based on active contour model: Active contour model is used to detect the top edge of the eyebrow. The eyebrow bounding box estimated from the position of the eye acts as an initial contour in the active contour model. This contour is then refined with every iteration. A maximum of 400 iterations are run. The Chan and Vese region-based energy model [5] is used to stop the contour on the eyebrow ridge line boundary. All the probabilities from the above three methods are added up and the line with the largest value is considered to be the superciliary edge of the eyebrow.

**Lips:** The last region of interest is the lips. To get the initial bounding box around the lips, the mouth cascade detector is used as suggested in CastrillÌon et al. [4]. The lips can be better segmented in HSV space rather than RGB space as the edges of the lips are well defined in HSV space. So, in the first step the video frame is converted from RGB to HSV space. Then the HSV space is binarized by thresholding H and S space using imbinarize function. If more than 48% of pixels were turned ON by the threshold operator in both H and S space, the intersection of the ON pixels in both H and S space is selected; otherwise, the ON pixels from the dominant space are selected. The binary image thus obtained is fed into bwareafilt MATLAB function. bwareafilt function labels different binary objects by size and picks the object with the largest size. In our case, the largest size corresponds to the lips. The binary mask thus obtained is then dilated with a spherical structuring element of size 7. This dilated mask acts as an initial contour for the active contour model which is then refined to obtain a smoother contour of the lips.

#### Tracking keypoints

**Fig. 1:**
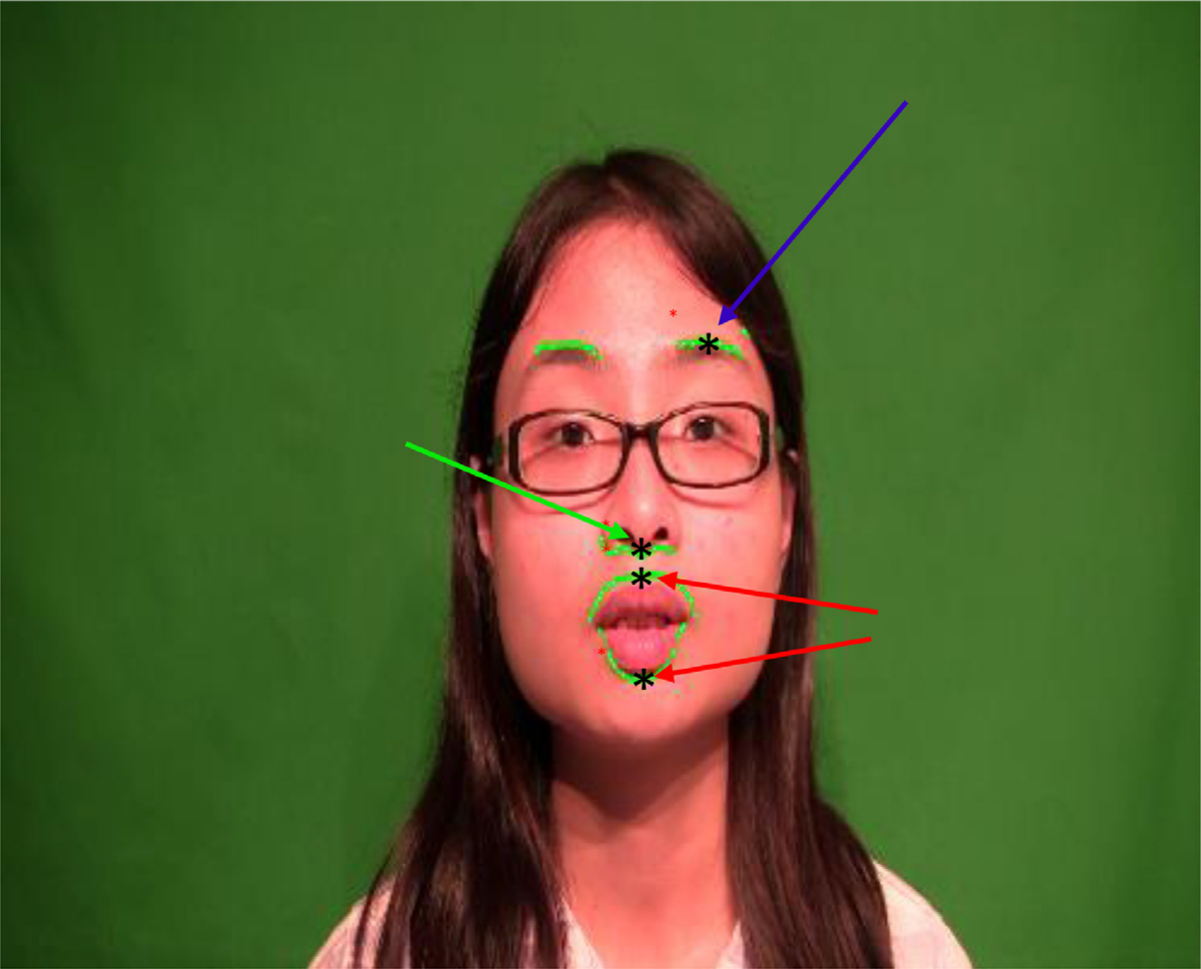
Detected points of interest (POI) for tracking in video.

The detection of ROIs is performed only on the first frame of the video. Instead of tracking all the detected points, representative points were selected from each ROI. These extracted representative keypoints are then tracked automatically in the video. The keypoints are decided based on where larger movements are expected. For the nose and eyebrow, the geometric means of the contours detected by the active contour model on the face, respectively, are selected as a keypoint. For the lips two keypoints are selected, one on the upper lip and another one on the lower lip. The upper keypoint is Cupid ’s bow corner of the lip and the lower keypoint is indicated by the center point between the two oral commissures. Fig. 1 shows all the selected keypoints that are tracked in the segmented video.

The tracking of the detected keypoints is performed using a feature tracking algorithm known as Kanade-Lucas-Tomasi (KLT) as proposed in Lucas et al. [13] and Tomasi et al. [18]. The tracking method is computationally efficient. It adopts a registration-based robust technique that uses intensity gradients to find a correspondence between two adjacent frames in the video. A set of motion trajectories for each keypoint is obtained. Translational head movement is subsequently removed from the eyebrow and the lips by subtracting the head displacements from the displacement of the eyebrow and lip keypoints.

#### Error analysis

In order to measure performance of the automatic method, we compared the detection and tracking results using manual annotation on a subset of data. A volunteer (not involved in the study) was presented with the image on a computer screen and was asked to trace out the curves on the left eyebrow, nose and the lips. MATLAB's *ginput.m* function was used to record the clicks of the mouse along the curve. To compare the results of the detection, the volunteer was presented with the first frame of one randomly sampled token from 14 subjects (9 females, 5 males). For tracking, we extracted frames #4, 8, 16, 64, and 128 from two randomly sampled video tokens: one of each gender. These were manually annotated as described above by the same volunteer.

The distance from the automatically detected POIs to each of the manually traced points on the curve was computed and the minimum Euclidean distance between them was considered an error. These errors were computed in pixels. Next, the computed errors were converted to percentages based on the image resolution of the videos (1080 × 1920). The errors are reported in Table 1 below.

**Table 1:** POI detection and tracking accuracy as estimated using human annotations created by a volunteer on randomly drawn tokens.

	Detection accuracy using 14 randomly drawn tokens Mean (SD) in %		Tracking accuracy using 2 randomly drawn tokensMean (SD) in%	
	Male	Female	Male	Female
Upper lip	0.71 (0.33)	0.86 (0.41)	0.45 (0.10)	0.77 (0.21)
Lower lip	0.68 (0.59)	0.49 (0.28)	0.22 (0.21)	0.49 (0.13)
Left eyebrow	0.54 (0.21)	0.61 (0.22)	0.64 (0.16)	0.44 (0.27)
Nose	0.47 (0.27)	0.50 (0.22)	0.49 (0.223)	0.24 (0.17)

Errors remain well below 1%, ranging from 0.22% to 0.86%, suggesting that our automatic landmark detection method is highly accurate and reliable.

#### Extracting features

**Fig. 2:**
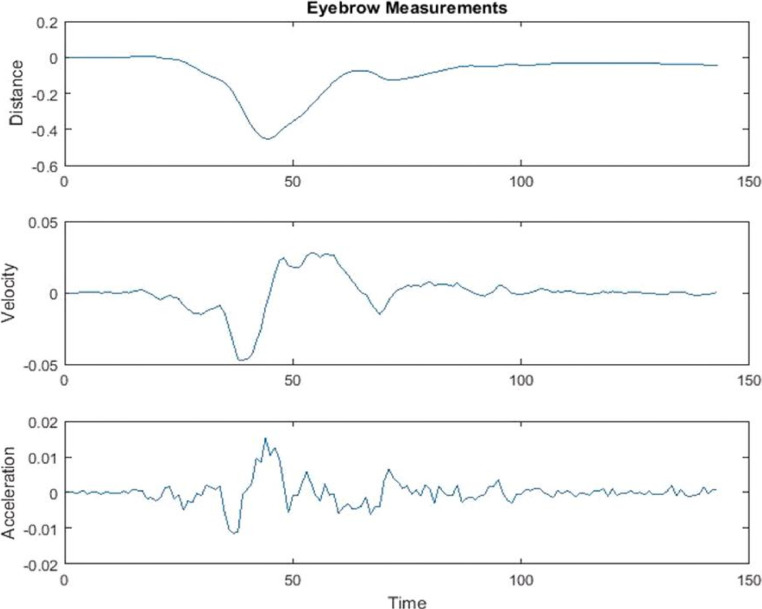
The measurements shown are normalized measurements (normalized to the head size).

Fig 2. shows an example of computed motion trajectories in a randomly selected video token. The top panel of Fig. 2 shows the distance of the keypoint from the first frame that is used as a reference frame. All motion is computed from the reference frame with the landmark points identified on the reference frame as origin. The middle and bottom panels of Fig. 2 correspond to velocity and acceleration, respectively. The velocity and acceleration are computed from the distance plot by taking the time derivatives. The derivatives are computed by taking the difference in measurement between the subsequent frames.

Once the keypoint tracking trajectories and their derivatives are computed, different features can thus be computed depending on what is of interest to the user. In the proposed method, we compute a number of features that summarize the trajectories obtained from each of the four tracked keypoints.

The computed features are categorized as distance-based, time-based and higher order kinematics-based. For the distance-based measures we capture the maximum distance traveled, minimum distance traveled and other statistics such as average and total distance traveled from the initial resting position. For time-based features, the relative time when the keypoint reached a maximum or minimum in the video token is measured. This is to capture whether the rising or lowering motion happened early on or later in the utterance. Lastly, the kinematic-based features are computed from the distance trajectories such as maximum velocity, minimum velocity and maximum acceleration during the utterance.

A summary of all the extracted features is listed in Table 2. These features were chosen to capture the different variations that could be introduced by the pronunciation of different Mandarin tokens used in Garg et al. [8]. (Table 3).

**Table 2:** The set of features used to represent each video token. ROI is a region of interest. Please refer to text for details.

ROI	Index	Category	How this feature is computed
Head	1	Distance	Maximum displacement of the head while head-raising from its starting position
Head	2	Distance	Maximum displacement of the head while head-lowering from its starting position
Head	3	Distance	Average distance head moved during the utterance
Head	4	Distance	Total distance traveled by head during the utterance
Eyebrow	5	Distance	Maximum displacement of the eyebrow keypoint from its starting position
Eyebrow	6	Distance	Maximum displacement of the eyebrow while eyebrow-lowering from its starting position
Eyebrow	7	Distance	Average distance eyebrow moved during utterance
Eyebrow	8	Distance	Total distance eyebrow moved during the utterance
Lips	9	Distance	Maximum lip-opening distance
Lips	10	Distance	Maximum lip-closing distance
Lips	11	Distance	Average distance lips moved during utterance
Lips	12	Distance	Total distance lips moved during the utterance
Head	13	Time	The relative time at which the displacement of the head while head-raising was maximum
Head	14	Time	The relative time at which the displacement of the head while head-lowering was maximum
Head	15	Time	The relative time at which the head velocity was maximum during head-raising
Head	16	Time	The relative time at which the head velocity was maximum during head-lowering
Eyebrow	17	Time	The relative time at which the displacement of the eyebrow while eyebrow-raising was maximum
Eyebrow	18	Time	The relative time at which the displacement of the eyebrow while eyebrow-lowering was maximum
Eyebrow	19	Time	The relative time at which the eyebrow keypoint reached maximum velocity during eyebrow-raising
Eyebrow	20	Time	The relative time at which the eyebrow keypoint reached maximum velocity during eyebrow-lowering
Lips	21	Time	The relative time at which the amount of lip-opening reached maximum
Lips	22	Time	The relative time at which the amount of lip-closing reached maximum
Lips	23	Time	The relative time at which the lip velocity during lip-opening was maximum
Lips	24	Time	The relative time at which the lip velocity during lip-closing was maximum
Head	25	Kinematic	Maximum head velocity during head-raising
Head	26	Kinematic	Maximum head velocity during head-lowering
Head	27	Kinematic	Maximum absolute acceleration of the head
Eyebrow	28	Kinematic	Maximum eyebrow velocity during eyebrow-raising
Eyebrow	29	Kinematic	Maximum eyebrow velocity during eyebrow-lowering
Eyebrow	30	Kinematic	Maximum absolute acceleration of the eyebrow
Lips	31	Kinematic	Maximum lip velocity during lip opening
Lips	32	Kinematic	Maximum lip velocity during lip closing
Lips	33	Kinematic	Maximum absolute acceleration of the lips

**Table 3:** Parameters that were used in the code at various steps.

	Code Listing	Parameters
Data		
	Audio sampling rate	16,000
	Video sampling rate	29.97 frames/s
Video Segmentation		
	Short Time Energy	window step size = 1
	Median Filter	Filter order = 5
	Clusters related to noise	threshold < 35% of maximum cluster length
Face		
	facedetector = vision.CascadeObjectDetector('FrontalFaceLBP', 'MergeThreshold',i);	
	Merge threshold	Merge threshold = 1 to 150 with increments of 10
		
Left eye	vision.CascadeObjectDetector('EyePairBig', 'MergeThreshold',a);	
	Merge threshold	Merge threshold = 0 to 150 with increments of 10
	vision.CascadeObjectDetector('LeftEye', 'MergeThreshold',i);	
	vision.CascadeObjectDetector('LeftEyeCART', 'MergeThreshold',i);	
	Merge threshold	Merge threshold = 4 to 150 with increments of 10
Eyebrow		
	Color probability threshold	0.2
	rgb2hsv(img);	
	edge(img(:,:,3),'canny');	
	activecontour(rgb2gray(img),mask,400);	iterations = 400
Nose	Merge threshold	Merge threshold = 1 to 150 with increments of 10
	activecontour(rgb2gray(img),mask,500,'edge','SmoothFactor',2);	iterations = 500
Lips	vision.CascadeObjectDetector('Mouth', 'MergeThreshold',i);	
	Merge threshold	Merge threshold = 4 to 150 with increments of 10
	rgb2hsv(img);	
	strel('sphere',7);	
	bwareafilt(lip,1);	Number of objects to keep = 1
	imdilate(bwconvhull(big),se);	spherical structuring element of radius = 7
	activecontour(rgb2gray(img),mask,500,'edge','SmoothFactor',2);	Number of iterations = 500
Random Forest	Trees	Number of trees = 500;
	Repeat sampling N times from the larger class	100
	mafdr(pval11,'BHFDR', true);	p value = 0.05
	*B* = TreeBagger(ntree,[*train*X *interaction*],trainY,'OOBPrediction','On', OOBPredictorImportance','on','MinLeafSize',20,'Method',' classification');	Random seed = 1 Min Leaf size = 20 OOBPrediction = on OOBPredictorImportance = on

In total, 33 features from the motion trajectories were extracted. Since the videos of the speakers were recorded from slightly variable distances from the camera, the features were first normalized to adjust this variance in order to run further statistical analysis. Furthermore, to compensate for different head sizes of the speakers, normalization was performed by dividing the obtained feature values by the shortest distance between the nose tip and the line joining the two eyes. The obtained feature values are measured in pixels and are also converted to the physical units (mm) by measuring the head size of the speaker in physical units.

**Fig. 3:**
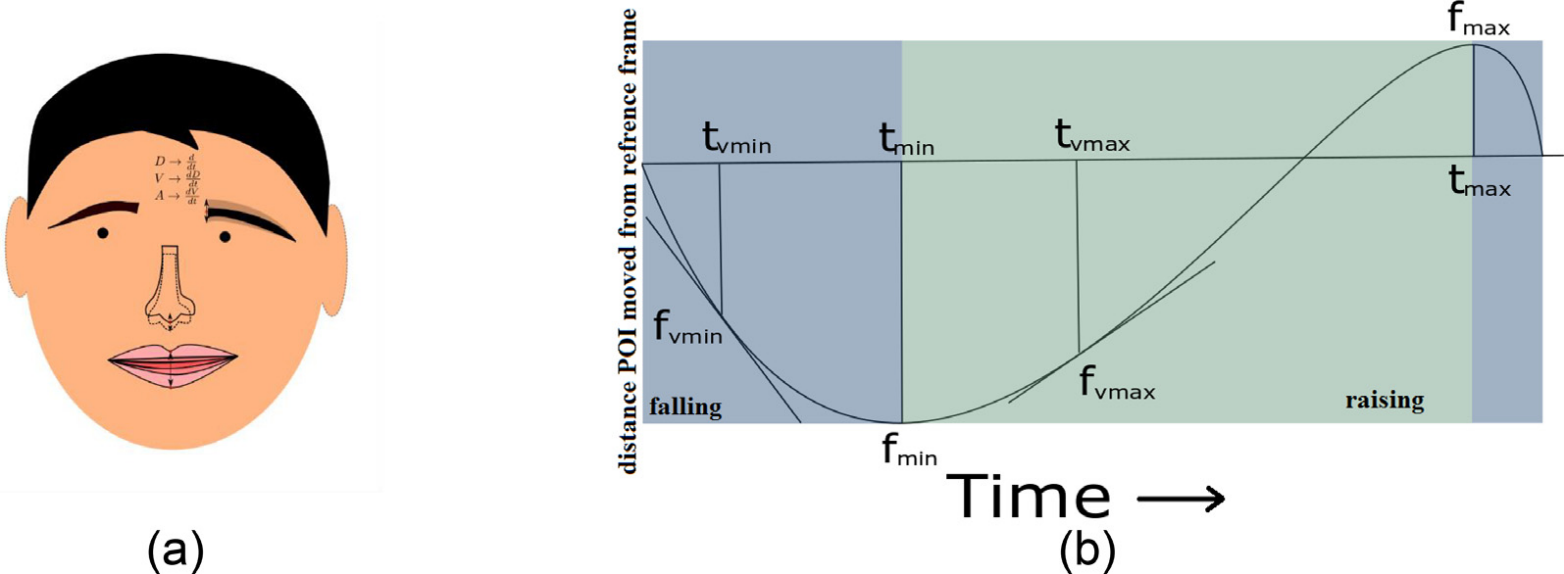
(a) Schematic diagram showing the position and direction of movement of the 4 tracked points. The shadows near eyebrow/jaw/nose indicate motion. (b) A visual summary of the different features we extracted from the 4 tracked keypoints. In (b), the blue regions mark the time instances when a tracked keypoint moved downward and hence represent the lowering movements of the head and eyebrow, or closing of the lips, while the green region represents rising movements of the head and eyebrow, or the opening of the lips. Note that velocity is indicated by the slopes of the curve (i.e., computed as rate of change of the curve) and the acceleration is computed by the rate of change in velocity.

Since the raising and lowering of pitch has been shown to be linked to the up and down articulatory movements [9,10], only the vertical movement of the keypoints is extracted and the features are measured in the vertical direction. Any movements in the horizontal direction are ignored in Garg et al. [8], but can be included in the analysis when needed. Fig. 3(a) shows a schematic diagram illustrating how the distance-based features and kinematic features are related. We also computed the relative time at which the minimum or maximum occurred with respect to the total duration.

### Two-part analyses of the extracted features

We used the classification of Mandarin tone to evaluate the extracted articulatory features. Mandarin has four distinct tones: high-level, mid-high rising, mid-low dipping and high-falling. In order to determine which features characterize each tone, a two-step analysis was performed. In the first step we identified which features differentiate each tone from the other tones by training a classifier. Once we found the significant features that characterize a tone, in the second step, we ran post-hoc analysis to compare the magnitude of the movement for that tone as compared to the other tones.

Since our problem consists of multiple classes (four classes corresponding to four tones), multi-class classification problem is transformed to binary class problem using one-versus-all (OVA) approach. In this approach, the features obtained from video tokens belonging to one tone are compared to features from all the other tones.

In our experiments, a random forest (RF) classifier is used to perform the classification tasks. RF was introduced by Breiman et al. [20] for binary and multi-class classification. An RF classifier consists of several decision trees that independently generate an output label given the features. Each tree is trained on a random subset of features using a random subset of data samples. The final label is assigned based on the majority vote of all the output labels by each of the trees. The random sampling of the features prevents the random forest from overfitting.

This approach was later extended by Paul et al. [15] to determine the significance of each feature toward classification. They showed that using only the significant features led to improvement in the performance compared to using all the features. The MATLAB implementation of RF provides a ranking of feature importance based on the feature weight derived from the training data. The significance is determined by evaluating the impact of each feature on classification performance, when the feature is randomly permuted. After training the RF classifier, each feature dimension was randomly permuted across the out-of-bag samples. Changes in the distribution of the class votes obtained by permuting a particular feature were then measured via a contingency table that summarizes the classification and misclassification rates (i.e. True Positive, True Negative, False Positive and False Negative) when the feature in question is permuted (or not). This procedure is repeated multiple times. A set of *p*-values were then obtained by running Pearson's χ^2^ test of independence on these measures using testcholdout MATLAB function. After corrections for multiple comparisons, features with adjusted *p*-values that are below the standard confidence level (*p* < 0.05) are henceforth regarded as “significant”.

Note that using the dataset with class labels of more than two classes as OVA classification in the same way as for a multi-class problem naturally leads to imbalanced classes. To address this, we employed bootstrapped sampling [12] so that the number of random samples (r) drawn for each class is the same. This step was repeated N times to eliminate bias towards any class.

In our experiments, we set *r* = 500 and set *N* empirically to 300 (we did not find any difference in classification performance when *N* > 100). We also employed *t* = 500 random trees for each tone classification task. Lastly, 90% of the samples in the entire dataset was used for training and 10% was used for testing in each of the sampling iterations.

## Software implementation and the application example

We present an algorithm to automatically detect salient regions on the face and track them in a video that can be later related to linguistic features such as tone in Mandarin Chinese. The software produced in this study was implemented in MATLAB (version 2017a). MATLAB provides a large collection of inbuilt libraries and is useful for quick prototyping. It contains several computer vision and audio processing related packages that can be used to extract meaningful features from the video data. The software code used in the study is made available on GitHub at: https://github.com/srbhgarg/avc.git. In the repository, avc_main.m is the main file that automatically reads all the MPEG-4 files present in the data directory and calls all the other functions to extract the features. Some samples of video data files are also provided in the GitHub repository in the data folder to test and play the code. The software uses native MATLAB functions such as VideoReader to read video data; audioread to read audio data; activecontour uses the Sparse-Field level-set method, similar to the method described in [3] for implementing active contour evolution. We used MATLAB's vision.PointTracker implementation of KLT to track the keypoints in the video.

TreeBagger function in MATLAB was used for Random Forest classifier. 500 trees were trained in the random forest with parameter MinLeafSize set to 20. MinLeafSize controls the minimum number of observations each leaf should have. This parameter controls the depth of the tree. The larger the number the shallower the tree will be. This in return reduces model complexity and computational time. In MATLAB, OOBPredictorImportance was also set so as to rate the different features in order of their importance for the classification task. Since random forest samples data randomly, for each feature separately, 50 different iterations were performed to get a robust estimate of the feature importance.

## Discussion

In this paper, we present a method and release software code that can be used to automatically detect and track landmark points on the face. Our work can be useful in research that involves the understanding of the movements of the facial landmarks made during speech production. The proposed pipeline's detection and tracking performances have error rates less than 1% of the image frame when evaluated using annotations created by a naive human annotator. Currently, our method can correct for the translational movements of the head. Other movements of the head such as rotations and tilts can be corrected using image registration (e.g. correct by registering each frame to a reference frame using external libraries such as OpenCV (https://www.learnopencv.com/head-pose-estimation-using-opencv-and-dlib/).

## Declaration of Competing Interests

The authors declare that they have no known competing financial interests or personal relationships that could have appeared to influence the work reported in this paper.
